# Damage Quantification Monitoring of CFRP Structures Cross-Area Based on Transfer Learning

**DOI:** 10.3390/s26165068

**Published:** 2026-08-10

**Authors:** Zhijun Liu, Haijun Zhang, Lijing Wang

**Affiliations:** 1 Key Laboratory of Civil Aviation Thermal Hazards Prevention and Emergency Response, School of Safety Science and Engineering, Civil Aviation University of China, Tianjin 300300, China; 2 School of Aeronautical Engineering, Beijing Polytechnic University, Beijing 100176, China

**Keywords:** carbon fiber reinforced polymer, structural health monitoring, cross-area, transfer learning, damage quantification

## Abstract

Given the widespread application of carbon fiber reinforced polymer (CFRP) structures in aerospace and automotive manufacturing, the critical role of structural health monitoring (SHM) is increasingly evident. Traditional damage quantification methods exhibit poor transferability and reusability in different structural areas, which are difficult to adapt to the complex and diverse damage characteristics of CFRP structures. To address this challenge, a multi-scale adaptive cross-area damage quantification method based on transfer learning (MSACADQ-TL) is proposed in this paper. Firstly, a multi-scale feature extraction framework is presented, which decomposes signals from various paths into multiple frequency domain sub-signals and analyzes the dynamic trends of these signals across different signal scales, providing richer damage feature information. Moreover, the difference between the source domain and the target domain is minimized to enhance transferability of features. Furthermore, this paper applies a damage quantification based on the Bidirectional Gated Recurrent Unit (BiGRU) model to achieve cross-area damage quantification. To validate the proposed method’s effectiveness, this paper designed three different cross-area damage transfer scenarios. Experimental results demonstrate that the MSACADQ-TL model outperforms other comparison methods in damage quantification tasks, which is effective for quantification damage in CFRP structures.

## 1. Introduction

Carbon fiber reinforced polymer (CFRP) composite structures have been extensively used in high-end equipment intelligent manufacturing, because of their exceptional specific strength, specific stiffness, and corrosion resistance [1,2,3]. However, CFRP structures inevitably develop various forms of structural damage under prolonged exposure to cyclic loading and multiple extreme environmental conditions, such as fiber breakage, matrix cracking, delamination, and interfacial debonding [4,5,6]. Consequently, accurately quantifying the damage size is crucial for preventing uncontrolled damage progression and avoiding sudden structural failure. Especially in critical fields like aerospace, early quantification of damage propagation is essential for ensuring the safety of CFRP structures [7,8].

Damages in CFRP structures, such as delamination or matrix cracks, are often from the interior, which are difficult to detect at an early stage. Furthermore, due to the complex laminated structure and anisotropy of CFRP materials, the precise quantification and assessment of damage propagation states become particularly challenging. Specifically, CFRP structures inherently possess anisotropic and inhomogeneous material properties due to variations in fiber orientation, layup sequence, resin distribution, and manufacturing process. Ultrasonic Lamb wave-based inspections of CFRP are considerably more challenging than those conducted on metals, owing to material anisotropy and numerous discontinuities arising from CFRP heterogeneity, layered construction, and potentially complex geometry. Even within the same CFRP panel, local variations in fiber volume fraction and ply stacking can lead to non-negligible differences in wave propagation characteristics, resulting in distinct signal distributions across different structural regions [9,10,11]. Furthermore, different structural areas may differ in terms of local geometry, thickness, stiffener configuration, and boundary conditions. The material anisotropy of CFRP laminates results in directionally dependent phase and group velocities, in addition to energy focusing, wave skewing, and beam spreading phenomena, which can lead to inaccurate damage assessment if not properly accounted for. These differences directly affect the wave propagation paths, reflection patterns, and mode conversion behaviors of Lamb waves, leading to systematic signal variations across structural regions even under identical damage conditions [12,13,14]. Additionally, the propagation path between the actuator and sensor varies across different structural areas, resulting in differences in wave travel distance, attenuation, and multipath interference, which introduce distribution shifts in the measured signals independent of the damage state itself [15,16]. Although non-destructive evaluation techniques can identify damage in CFRP components, contemporary inspection methodologies face substantial constraints including excessive operational costs, limited accessibility to hidden or internal damage regions, and considerable dependency on inspector expertise and interpretation. These limitations hinder accurate quantification of damage severity in composite structures [17,18].

Structural health monitoring (SHM) technology is gradually becoming an important way for structure damage quantification [19,20]. Among various SHM techniques, Lamb wave-based methods utilizing integrated piezoelectric sensors have gained widespread adoption throughout the entire lifecycle of structures to maintenance [21]. However, the structural damage forms of CFRP composite materials are complex, the damage types are diverse, and the service environment is harsh [22]. The existing SHM technology has problems such as poor accuracy and poor robustness in the damage quantification results during damage monitoring. And it poses important challenges to achieving real-time health monitoring and early damage detection in CFRP structures [9].

Machine learning has emerged as the cornerstone technology for intelligent real-time damage monitoring in CFRP structures, offering unprecedented capabilities in automated damage assessment and prognostics [23,24]. Khatir et al. [25] proposed a method for assessing damage in laminated composite plates based on Cornwell Indication, Artificial Neural Networks (ANNs), and Particle Swarm Optimization. Saadatmorad et al. [26] developed a method utilizing wavelet transforms and ANNs for damage quantification in rectangular laminated composite plates. In addition to traditional machine learning methods, machine learning methods mainly based on deep learning have also been applied in CFRP damage monitoring. Helwing et al. [27] employed in situ X-ray CT analysis combined with a Convolutional Neural Network (CNN)-based fault segmentation approach to analyze the damage evolution in GFRP composites. Zhou et al. [28] proposed an infrared thermography-based method using Attention U-Net for CFRP surface defect characterization in rapid and accurate defect detection. These machine learning methods require high-quality data, especially for damage propagation data. However, obtaining a large amount of high-quality damage data is difficult and costly. Particularly in practical applications, damage data in CFRP structures do not uniformly appear in different areas of the structure. Moreover, due to the complex multi-layered and anisotropic characteristics of CFRP structures, there are significant differences in Lamb wave signal distributions across various structural areas, even when the damage sizes are identical. This is because Lamb wave signals are jointly influenced by material heterogeneity, geometric boundary conditions, and propagation path variations across different structural regions. Consequently, if a machine learning model trained in one area is directly applied to another, the model’s generalization performance may be insufficient, leading to inaccurate damage quantification. In large-scale CFRP structures such as aircraft panels or wind turbine blades, obtaining sufficient labeled training samples from every structural region of interest is practically infeasible due to cost, time, and accessibility constraints [29,30], which further compounds the cross-area generalization challenge.

Transfer learning (TL), by leveraging data and models from other similar tasks, can still perform well in situations where data is scarce [31,32]. Gulsen et al. [33] introduced the UNDT dataset comprising 1150 ultrasonic images from 60 composite materials and demonstrates that transfer learning methods in automated defect detection, significantly outperforming traditional inspection approaches. Wang et al. [34] introduced an adaptive damage monitoring approach utilizing transfer learning techniques to achieve integrated capabilities in damage existence detection, damage type identification, and spatial damage localization. For key areas in CFRP structures where it is difficult to obtain sufficient data, TL allows models to transfer knowledge from data-rich areas to improve the damage quantification performance in data-scarce areas, which is of significant importance for SHM in practical engineering applications. Although transfer learning has achieved remarkable success in the field of SHM, its application to CFRP damage quantification remains unexplored, particularly lacking research on cross-region damage quantification under small-sample conditions. This technological gap constrains the advancement of CFRP structural health monitoring, as cross-region damage quantification technology possesses tremendous potential. It can effectively leverage existing damage data and models from monitored regions to rapidly extend monitoring capabilities to new areas, thereby enabling comprehensive monitoring of critical structural components and early warning of potential safety hazards. Therefore, this paper proposes a TL-based cross-area damage quantification method that achieves damage size prediction in different scenarios. The specific contributions are as follows.

(1) A multi-scale damage feature extraction framework is constructed. This framework not only provides a refined decomposition of signals in the frequency domain but also expresses damage features in the time domain, enabling multi-dimensional feature representation of signals on both temporal and frequency scales. It extracts intrinsic multi-level features from damage data and maps transferable damage features through feature mapping.
(2) A cross-area damage quantification transfer learning method is proposed. Focusing on a small-sample damage dataset, this method establishes a correlation between damage size and multi-dimensional features, achieving damage quantification assessment in multiple scenarios.
(3) The combination of manual feature extraction methods with deep neural networks is employed to further refine damage-sensitive features, thereby enhancing the model’s expressive capacity for damage features. This method can effectively learn damage features with limited labeled data to accurately quantify damage size and achieve cross-area damage quantification without the need for samples from new damage areas.

The structure of this paper is as follows: Section 2 elaborates on the proposed method; Section 3 provides a detailed description of the experimental setup; Section 4 offers a thorough analysis of the results; and Section 5 draws the conclusion of this paper.

## 2. Method Introduction

**Figure 1:**
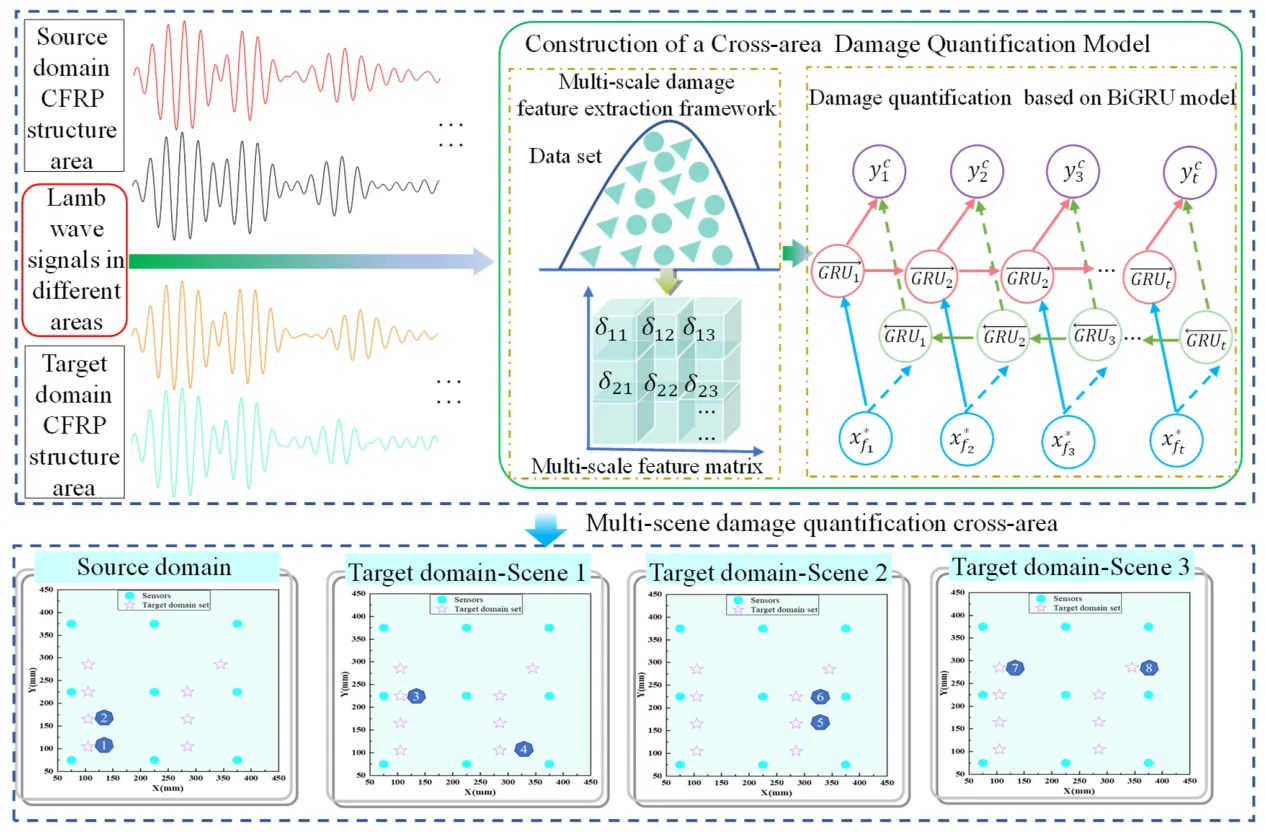
Schematic diagram of the cross-area damage quantification method.

In the field of practical engineering applications of CFRP structures, there is a significant challenge due to the scarcity of effective data and the limitation of damage samples, which severely restricts the accuracy of damage quantification results. Achieving precise cross-area damage quantification with limited samples is a technical challenge that urgently needs to be addressed. This paper combines feature extraction technology with deep learning models to propose a method for cross-area damage quantification called MSACADQ-TL. The specific technical process is detailed in Figure 1. The proposed methodology is structured as outlined below. Firstly, a multi-scale damage feature extraction framework is developed, in which raw signals acquired from multiple sensing paths are decomposed into sub-signals across diverse frequency domains. The dynamic trends of these sub-signals are subsequently analyzed at varying scales, thereby yielding a comprehensive and enriched representation of damage-relevant features. Then, to mitigate the distributional discrepancy between the source domain and the target domain, a domain adaptation strategy is incorporated into the framework. By minimizing the inter-domain divergence, the transferability and generalizability of the extracted features are substantially enhanced. Finally, building upon the transferred features, a damage quantification model based on the BiGRU architecture is constructed to achieve accurate cross-area damage quantification.

### 2.1. Multi-Scale Damage Feature Extraction Framework

This paper proposes a multi-scale damage feature extraction method aimed at enhancing the transferable ability of the same damage size in different areas. Considering the nonlinear and non-stationary characteristics of Lamb wave signals, this paper introduces Variational Mode Decomposition (VMD), an adaptive non-recursive signal processing technique [35,36,37], to decompose complex signals into a finite number of sub-signals with different central frequencies, thereby extracting richer damage feature information from various frequency bands. In this study, the number of decomposition modes in VMD is set to 3, meaning that each Lamb wave signal is decomposed into 3 sub-signals with distinct central frequencies. This selection was determined through preliminary experiments, which demonstrated that 3 modes provide a stable and representative decomposition of the Lamb wave signals while the variation in the number of modes has a relatively limited influence on the final damage quantification performance. Compared with the traditional empirical mode decomposition method, VMD has a solid theoretical foundation. First, a variational function is constructed, as shown in Equation (1). Then this function is solved, and the specific solving principles are detailed in Equation (2).

(1) $$\underset{\left\{u_{k}\right\}\left\{\omega_{k}\right\}}{\min}\left\{\sum_{k}{\left\|\partial_{t}\left[\left(\delta \left(t\right)+\frac{j}{\pi t}\right)u_{k}\left(t\right)\right]e^{-j\omega_{k}t}\right\|}_{2}^{2}\right\}\;s.t.\sum_{k}u_{k}\left(t\right)=L\left(t\right)$$

(2) $$L\left(\left\{u_{k}\right\},\left\{\omega_{k}\right\},\lambda \right)=\alpha \sum_{k}{\left\|\partial_{t}\left[\left(\delta \left(t\right)+\frac{j}{\pi t}\right)u_{k}\left(t\right)\right]e^{-j\omega_{k}t}\right\|}_{2}^{2}+\;\|L^{*}\left(t\right)-\sum_{k}u_{k}\left(t\right){\|}_{2}^{2}+\langle \lambda \left(t\right),L^{*}\left(t\right),\sum_{k}u_{k}\left(t\right)\rangle$$

where {$u_{k}$} denotes the set of *k* sub-signals, with *k* representing the number of signals. {$\omega_{k}$} represents the set of central frequencies. $L^{*}\left(t\right)$ signifies the Lamb wave signal to be decomposed. *λ*(*t*) is the Lagrange multiplier operator, and *α* is the quadratic penalty factor.

Subsequently, multi-scale permutation entropy (MPE) [38] is utilized to analyze each sub-signal, measuring the trend of signal changes and capturing the dynamic variations and complexity of signals across different scales. Firstly, the Lamb wave sub-signals are coarsened according to Equation (3) to capture multi-scale signals. Then the coarse-granulated sequences are reconstructed and sorted in ascending order, and the frequency of occurrence $P_{r}$ for each sequence is statistically analyzed. Finally, the PE of each coarse-granulated sequence is calculated, as shown in Equation (4), where *h* is the scale factor, *γ* is an integer, *R* ≤ *m*! and *m* represents the embedding dimension used in the reconstruction process.

(3) $$y_{\gamma }^{h}=\frac{1}{h}\sum_{t=\left(\gamma -1\right)h+1}^{\gamma h}u_{k}\left(t\right)$$

(4) $$PE(m)=-\sum_{r=1}^{R}P_{r}\ln P_{r}$$

Furthermore, this paper maps the damage data from the source domain structural area and the target domain structural area into a high-dimensional Reproducing Kernel Hilbert Space (RKHS) [39]. Within this space, a low-dimensional embedding is sought, and the Maximum Mean Discrepancy (MMD) [40] distance is optimized to minimize the distribution difference between the source and target domain data. The detailed derivation process can be found in Reference [40].

### 2.2. Damage Quantification Based on the BiGRU Model

**Figure 2:**
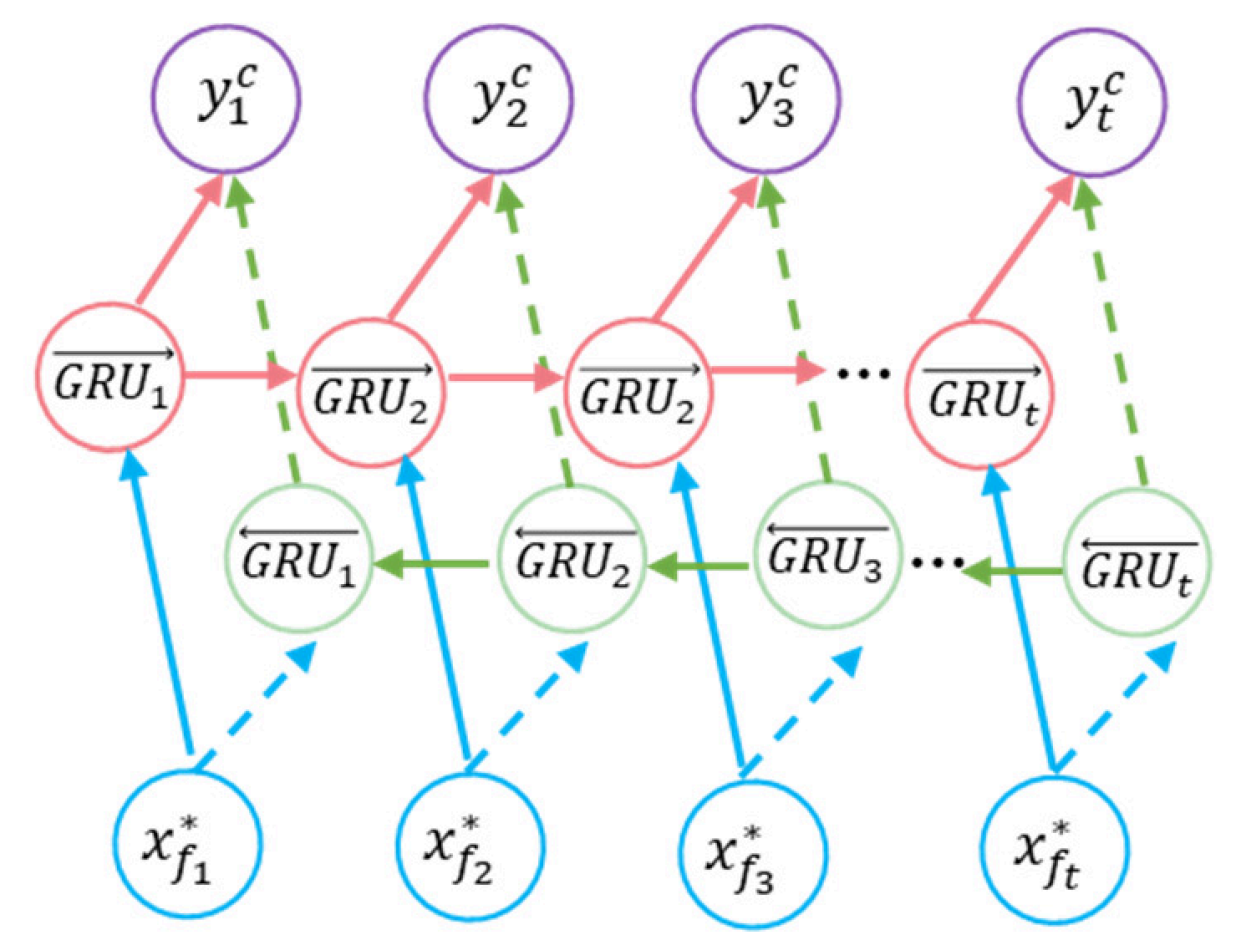
BiGRU model structure.

BiGRU is an extension of the Gated Recurrent Unit (GRU), which combines GRUs in both forward and reverse directions [41]. As depicted in Figure 2, the structure of the BiGRU model allows it to capture information from both forward and backward propagation, so that more important features can be fully used and the features extracted by the network are more abundant. BiGRU effectively handles long-term dependencies in time series through a gating mechanism that captures temporal dependencies in structural response data. This is critical for damage detection and quantification, as structural damage tends to exhibit specific pattern changes over time series.

### 2.3. Multi-Scale Adaptive Cross-Area Damage Quantification Method Based on Transfer Learning

The MSACADQ-TL method proposed in this paper utilizes a multi-scale damage feature extraction framework to effectively capture transfer features in the data that represent damage information; meanwhile, the deep learning model based on BiGRU is capable of automatically learning and recognizing complex and abstract features in the data. This method provides more comprehensive and rich damage representation information, thereby significantly improving the accuracy of damage quantification. The specific procedures are described as follows.

The first step is signal preprocessing. The Lamb wave signals collected from the damage area of the source domain CFRP plate are denoted as $L_{s}\left(t_{i}\right)$, and the signals collected from the damage area of the target domain CFRP plate are denoted as $L_{T}\left(t_{j}\right)$. After data preprocessing, the normalized signal datasets are obtained as $L_{S}^{*}\left(t_{\xi }\right)$ and $L_{T}^{*}\left(t_{\eta }\right)$.

The second step is the construction of a multi-scale damage feature extraction framework. Under the premise of limited samples, enriching feature information to expand the amount of information is the focus of this step. Among the many algorithms for enriching signal features, VMD, as an adaptive signal processing method, can decompose complex signals into a finite number of sub-signals with different central frequencies [27], which can better represent the damage features of the signals from different frequency domain dimensions. The source domain signal $L_{S}^{*}\left(t_{\xi }\right)$ is decomposed into sub-components $u_{s}\left(t_{i}\right)$ using the VMD method, and the target domain signal components are $u_{t}\left(t_{j}\right)$. The specific derivation equations are in Equations 1,2. Each sub-signal is integrated with the MPE principle to further characterize the underlying damage-sensitive features inherent in the decomposed frequency components [38]. The component signals are subjected to coarse-graining analysis to obtain the source and target domain’s coarse-grained sequences ${\left.y_{S}\right|}_{i}^{h}$ and ${\left.y_{T}\right|}_{j}^{h}$. Further, their respective multi-scale permutation entropy matrices are calculated as follows: $M_{S}\;=\;\left[{\mathrm{PE}}_{S}\left(1\right),{\mathrm{PE}}_{S}\left(2\right),\ldots {\mathrm{PE}}_{S}\left(l\right)\right]$ and $M_{T}\;=\;\left[{\mathrm{PE}}_{T}\left(1\right),{\;\mathrm{PE}}_{T}\left(2\right),\ldots {\mathrm{PE}}_{T}\left(l\right)\right]$. These feature matrices, which can reflect damage information from the source and target domains, are mapped into the RKHS to find transfer components. The MMD is used as a measure to learn common features across domains and obtain a migratory matrix that can reflect damage: $M_{S_{-}final}\;=\;\left[{PE}_{S}^{*}(1),{PE}_{S}^{*}\left(2\right),\ldots {PE}_{S}^{*}\left(m\right)\right]$ and $M_{T_{-}final}\;=\;\left[{PE}_{T}^{*}(1),{PE}_{T}^{*}\left(2\right),\ldots {PE}_{T}^{*}\left(n\right)\right]$.

The third step involves the construction of a damage quantification model that combines multi-scale damage transfer features with the BiGRU method. This approach can effectively learn and train Lamb wave signals, thereby fully extracting the proposed features even with a limited sample size. The damage quantification model, once trained in the source domain CFRP structure, can be directly used to the target domain without the need for additional fine-tuning, thus achieving good performance in the target domain. The test data is denoted as $M_{S_{-}final}$, with test labels representing seven different sizes of damage, which are input into the BiGRU model. The BiGRU is capable of learning the damage propagation relationship from Lamb wave data, which is crucial for damage quantification, as the development of structural damage is often related to long-term environmental effects and material degradation [42]. The number of hidden units of the model is 5, the initial learning rate is 0.1, and the maximum number of epochs is 200. Adam algorithm is selected as the Optimizer of the model.

## 3. Experimental Setup

In order to verify the effectiveness of the proposed damage quantification algorithm, three experiments are designed and conducted to evaluate the performance of the proposed method by quantifying the damage in three different areas of the CFRP structure.

### 3.1. Experimental Configuration Based on CFRP Composite Plate Structure

**Figure 3:**
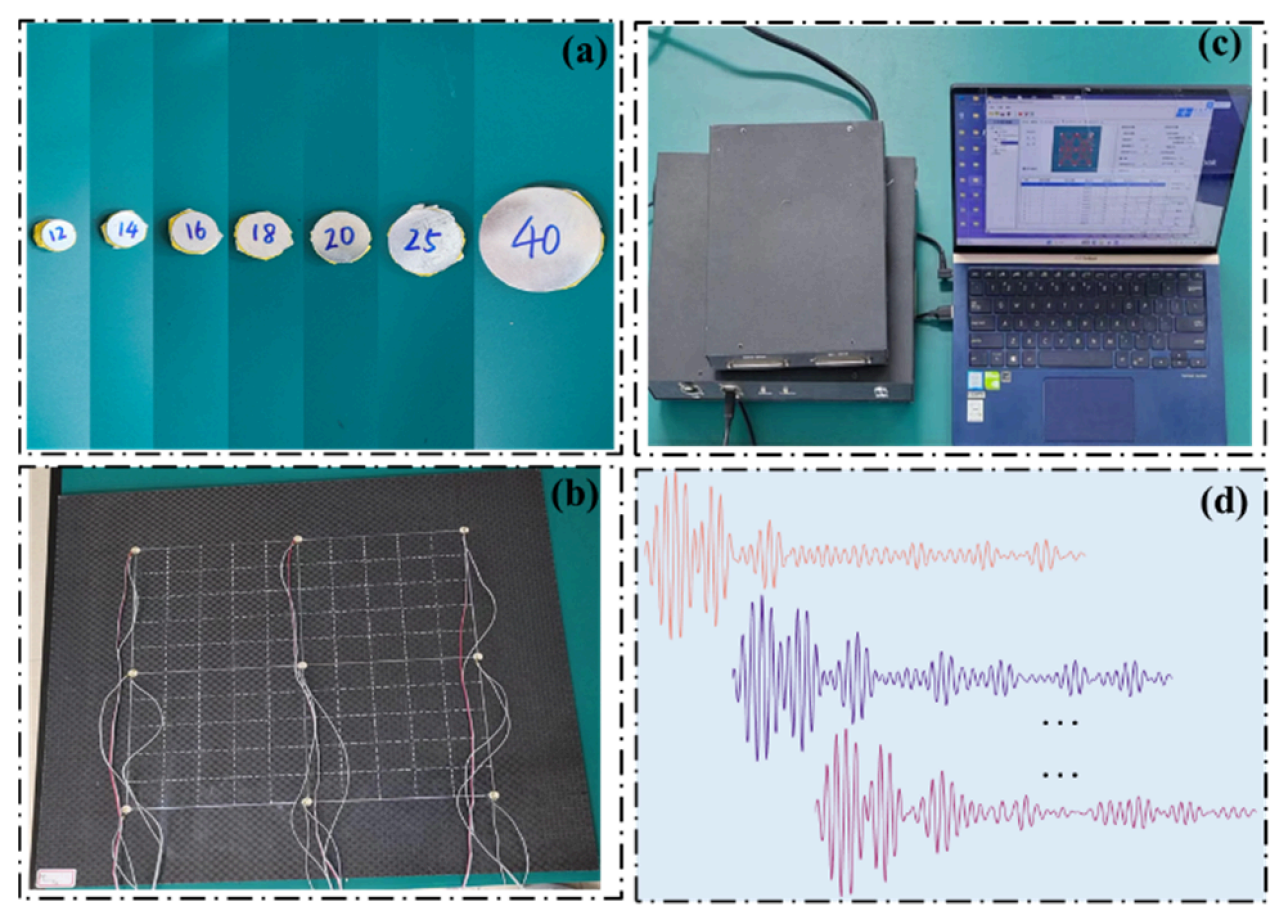
Construction of cross-area damage quantitative monitoring experimental system. (**a**) Simulated damages; (**b**) monitoring area with PZTs; (**c**) Lamb wave signal acquisition system; (**d**) Lamb wave signals.

In the experiment, absorbing wave material is used to simulate damages of different diameters. While simulated and actual damages are not entirely equivalent, prior studies have shown [43] that damages simulated by attaching absorbing wave material to the structure can cause asymmetry in structural stiffness and absorption of wave energy. This simulation method can cause a reduction in amplitude and changes in flight time, effects that are similar to those produced by real damages [44]. The specific sizes are shown in Figure 3a, where seven different diameters (d = 12 mm, 14 mm, 16 mm, 18 mm, 20 mm, 25 mm, 40 mm) and a thickness of 2 mm of absorbing wave material is attached to the surface of the specimen to simulate different artificial damages. For this chapter, a CFRP composite plate structure with dimensions of 450 mm × 450 mm × 3 mm is selected as the experimental specimen. The CFRP plate is composed of six layers of T700 12K carbon fiber woven cloth, and these layers are arranged in the sequence of ${\left[0{}^{\circ}/90{}^{\circ}\right]}_{3}$, as shown in Figure 3b. The damage monitoring area is shown in Figure 3b. The Lamb wave signal acquisition system is depicted in Figure 3c. A narrowband five-peak sine signal modulated by a Hanning window was used as the excitation, with the excitation frequency set to 80 kHz and a sampling rate of 24 MHz. The output signal’s peak voltage was configured at 60 V, with the gain adjusted to 40 dB. The damage signals obtained through the Lamb wave information acquisition system shown in Figure 3c are depicted in Figure 3d.

### 3.2. Lamb Wave Data Acquisition

**Figure 4:**
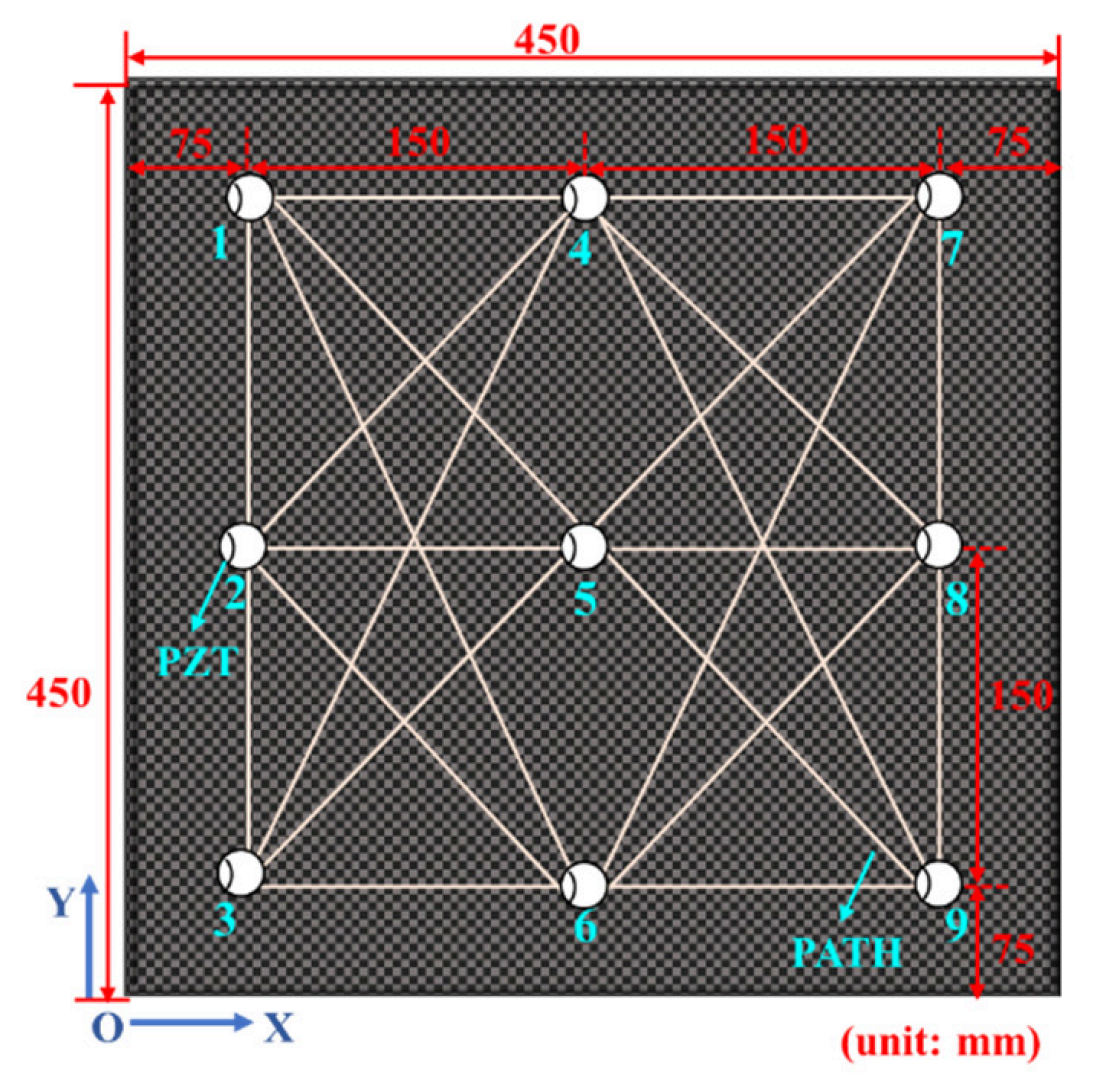
Circular PZT-5A sensor layout.

In this experiment, a total of 9 uniformly sized circular PZT sensors are used to collect Lamb wave signals in this chapter. The specific layout of these sensors is detailed in Figure 4. The diameter of each PZT sensor is 8 mm, with a thickness of 0.48 mm. Among them, sensors numbered 4, 5, and 6 were used as actuators, while the remaining sensors served as receivers. Within the entire sensing network, a total of 22 sensing paths were established to achieve comprehensive monitoring of the composite material structure.

**Figure 5:**
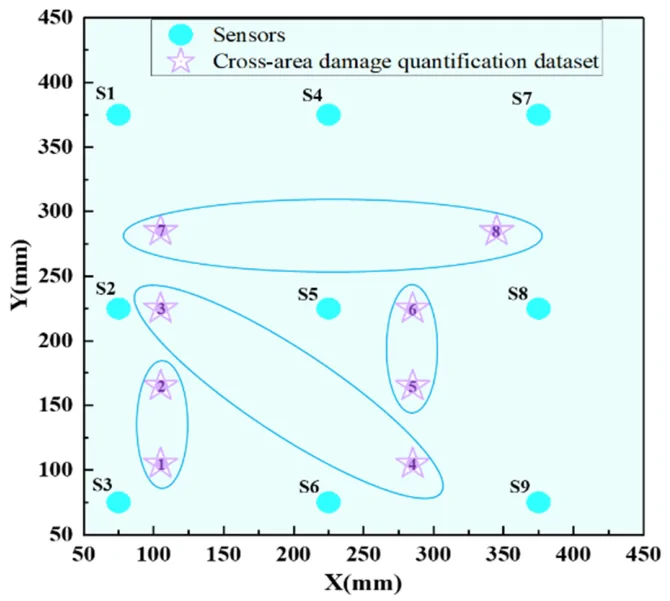
Schematic diagram of the damage expansion area.

In this experiment, 8 positions were randomly selected on the CFRP plate shown in Figure 3b. These locations are divided into 4 distinct areas, each consisting of two locations. Each distinct area is demarcated by a blue elliptical boundary, as illustrated in Figure 5. Specifically, the areas annotated with the numerals 1 and 2 within these elliptical delineations are designated as the source domain damage areas. The remaining three elliptical areas serve as the target domain damage areas. Consequently, three distinct damage transfer quantification scenarios are established, which are detailed in Table 1.

To reduce measurement noise and enhance the signal-to-noise ratio (SNR), this study employed a strategy of repeated signal sampling. Specifically, the Lamb wave signals for each transfer scenario were independently collected three times, with each collection carried out under identical conditions to ensure data consistency and comparability. Then the signal data obtained from these three times is averaged to eliminate the influence of random errors and noise to obtain a more stable and reliable final data set.

**Figure 6:**
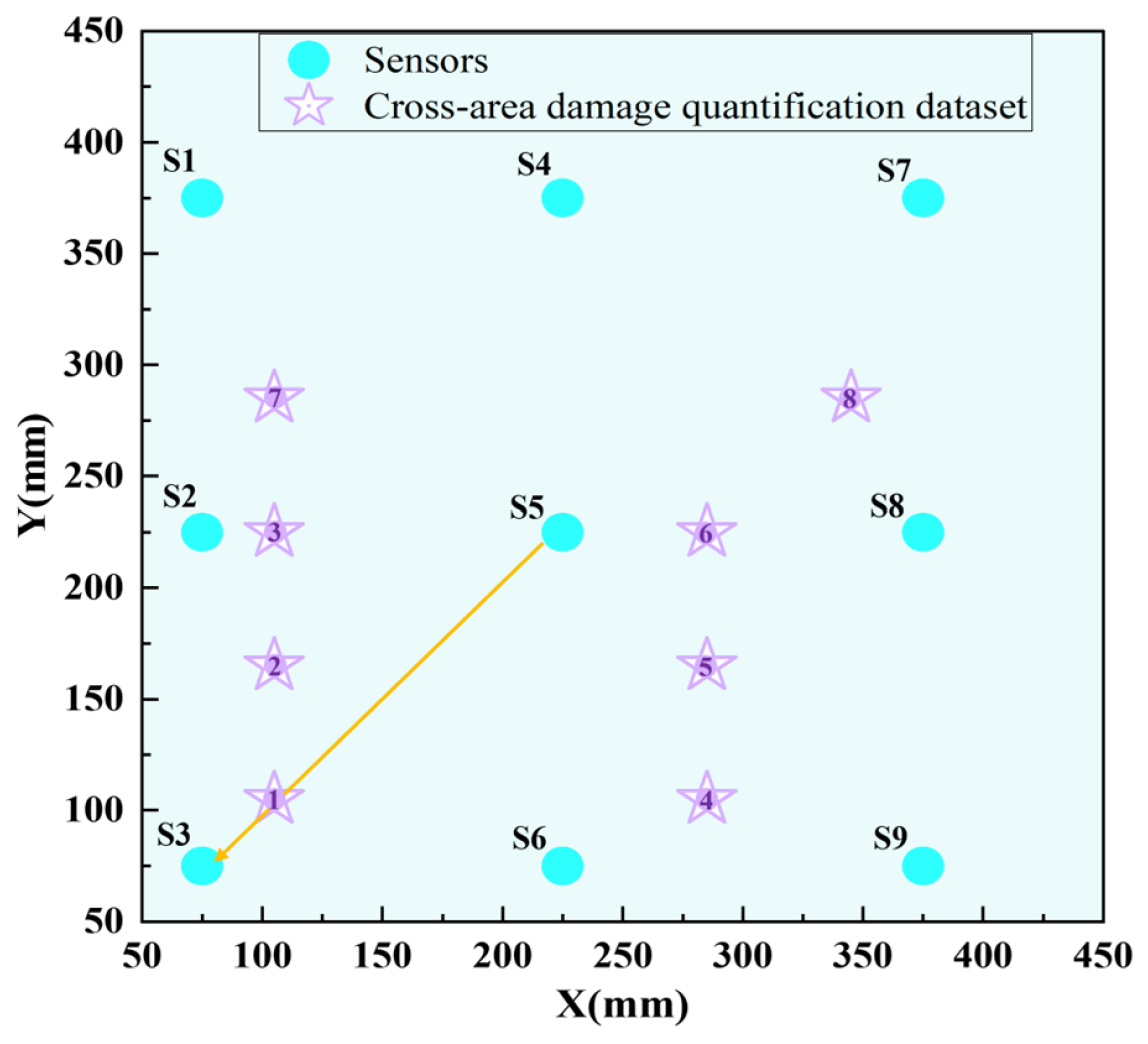
Sensing path under damage location 1.

**Figure 7:**
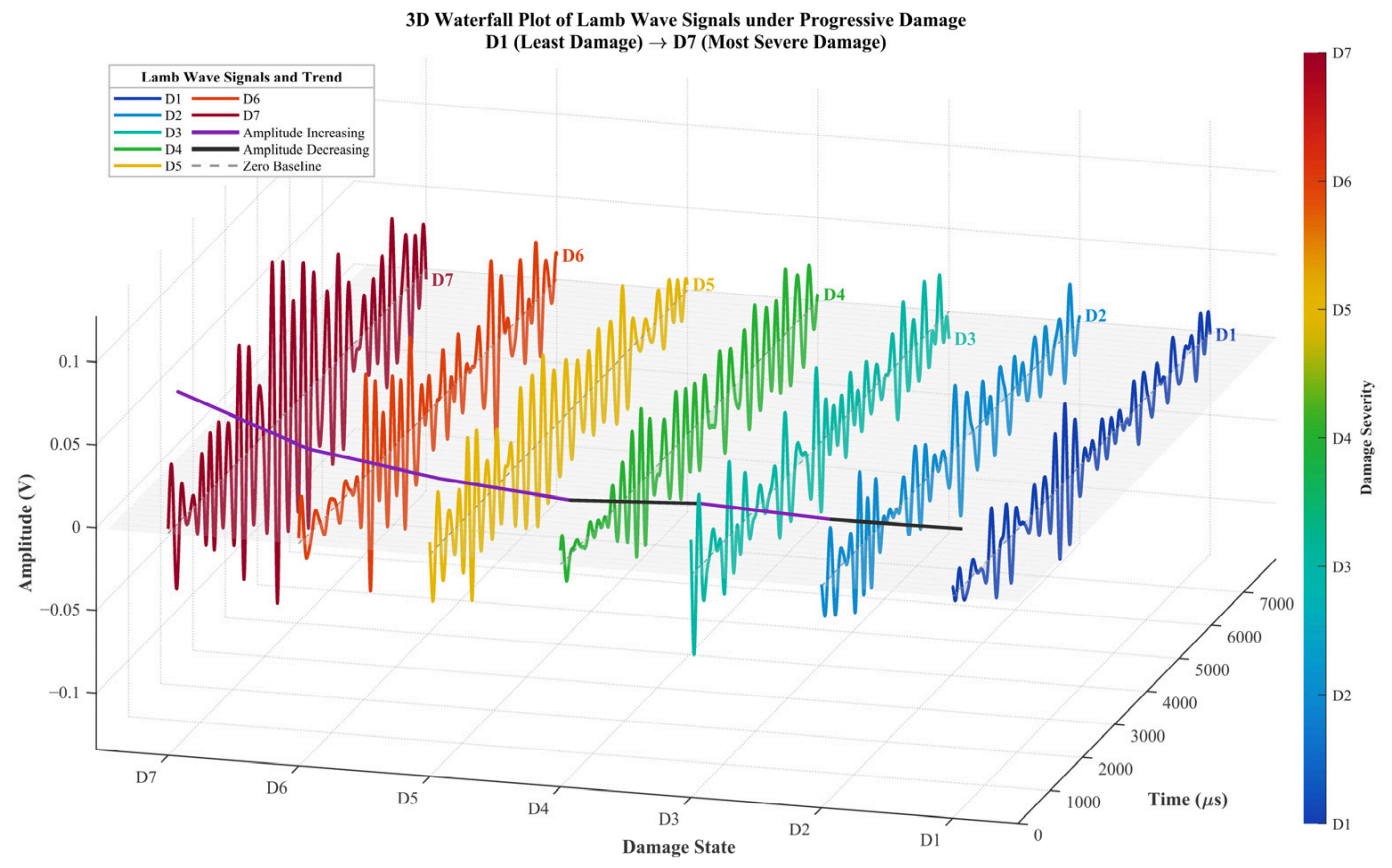
Distribution of scattering signals for seven different damage sizes at the same location.

To investigate the influence of damage progression on Lamb wave signal characteristics, seven stages of damage extension at Position 1 (as labeled in Figure 6) are examined, with the sensor path indicated by the arrow in Figure 6 selected as a representative example. The corresponding scattered Lamb wave signals for the seven damage states (D1–D7) are presented in Figure 7.

As evidenced by Figure 7, a positive correlation exists between signal amplitude and damage size: the peak-to-peak amplitude exhibits an overall upward trend from D1 to D7, with the rate of increase becoming markedly more pronounced beyond a damage size of 20 mm (i.e., from D5 onward). This observation indicates that larger damage dimensions give rise to stronger scattered wave energy, attributable to the intensified interaction between the propagating Lamb waves and the expanding damage boundary. Furthermore, a progressive increase in waveform complexity is observed with increasing damage severity. In particular, the signals corresponding to D6 and D7 not only exhibit elevated amplitudes but also display substantially more complex waveform morphologies, characterized by the emergence of higher-frequency components and more pronounced oscillatory envelopes. This phenomenon reflects the increasingly significant perturbation imposed by the growing damage on the Lamb wave propagation path, resulting in enhanced signal non-stationarity that further corroborates the sensitivity of Lamb wave-based monitoring to damage progression.

## 4. Experimental Case Results and Discussion

The damaged area marked with the numbers 1 and 2 in Figure 5 is utilized as the source domain data to train the transfer learning model. Data from the remaining three damaged areas are employed as the target domain samples. The damage quantification results in the target area were verified without the need to fine-tune the model.

### 4.1. Damage Quantification Results Based on MSACADQ-TL Model

**Figure 8:**
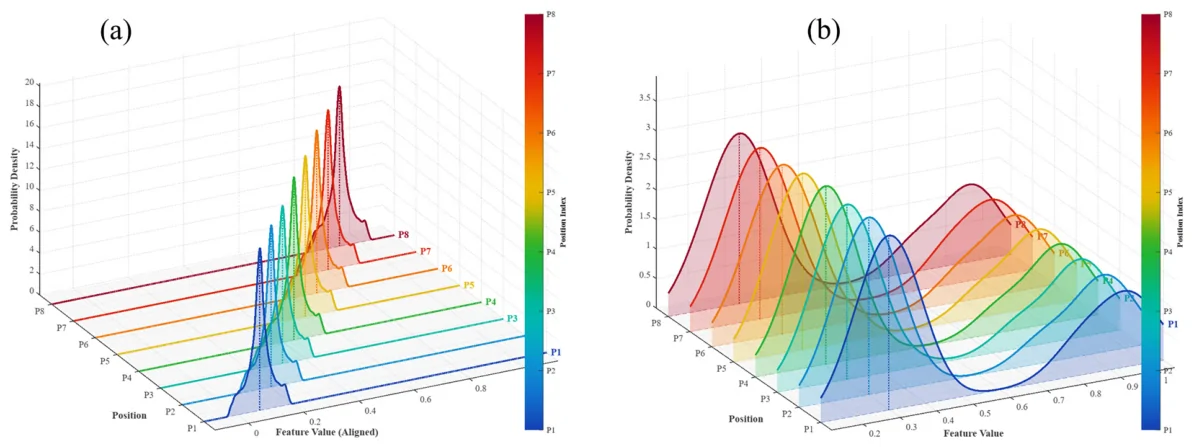
Probability density distributions of the same damage at eight positions before and after transfer learning: (**a**) feature distribution before transfer learning; (**b**) feature distribution after transfer learning.

To further validate the domain adaptation capability of the proposed framework, Figure 8 presents visualizations of the source domain and target domain (transfer scenario 1) feature distributions before and after transfer learning, illustrating the evolution of signal representations across eight measurement positions for the 12 mm damage case.

Prior to transfer learning in Figure 8a, the probability density distributions of signals acquired at the eight measurement positions exhibit sharply peaked, narrow-bandwidth characteristics with pronounced inter-class separability. This pattern indicates that positional factors dominate the original feature space, rendering the learned representations strongly position-dependent. Consequently, the model’s generalization capability in cross-region scenarios is fundamentally constrained, as the extracted features reflect spatial measurement conditions rather than intrinsic damage-related information.

Following the application of the proposed transfer learning framework in Figure 8b, the probability density curves corresponding to all eight measurement positions converge toward broad, overlapping distributions within an approximately unified probabilistic subspace. This convergence demonstrates that the proposed method effectively suppresses the confounding influence of measurement position on feature representation and successfully establishes domain-invariant feature embeddings.

In this paper, three damage datasets under different transfer scenarios are applied to evaluate the quantitative performance of the proposed cross-area transfer damage diagnosis model. The mean absolute error (MAE) [45] and root mean square error (RMSE) [46] were selected as the key metrics for damage quantification assessment. In both of these quantification metrics, a lower value indicates better quantitative performance of the model. Therefore, the low values of MAE and RMSE are a direct reflection of the prediction accuracy of the model, and they together provide a solid theoretical basis for evaluating the quantitative performance of the cross-area transfer damage diagnosis model. The specific equations are shown in Equations (5) and (6).

(5) $$MAE=\frac{1}{k}\sum_{i=1}^{k}\left(y_{i}-{\hat{y}}_{i}\right)$$

(6) $$RMSE=\sqrt{\frac{1}{k}\sum_{i=1}^{k}{\left(y_{i}-{\hat{y}}_{i}\right)}^{2}}$$

**Figure 9:**
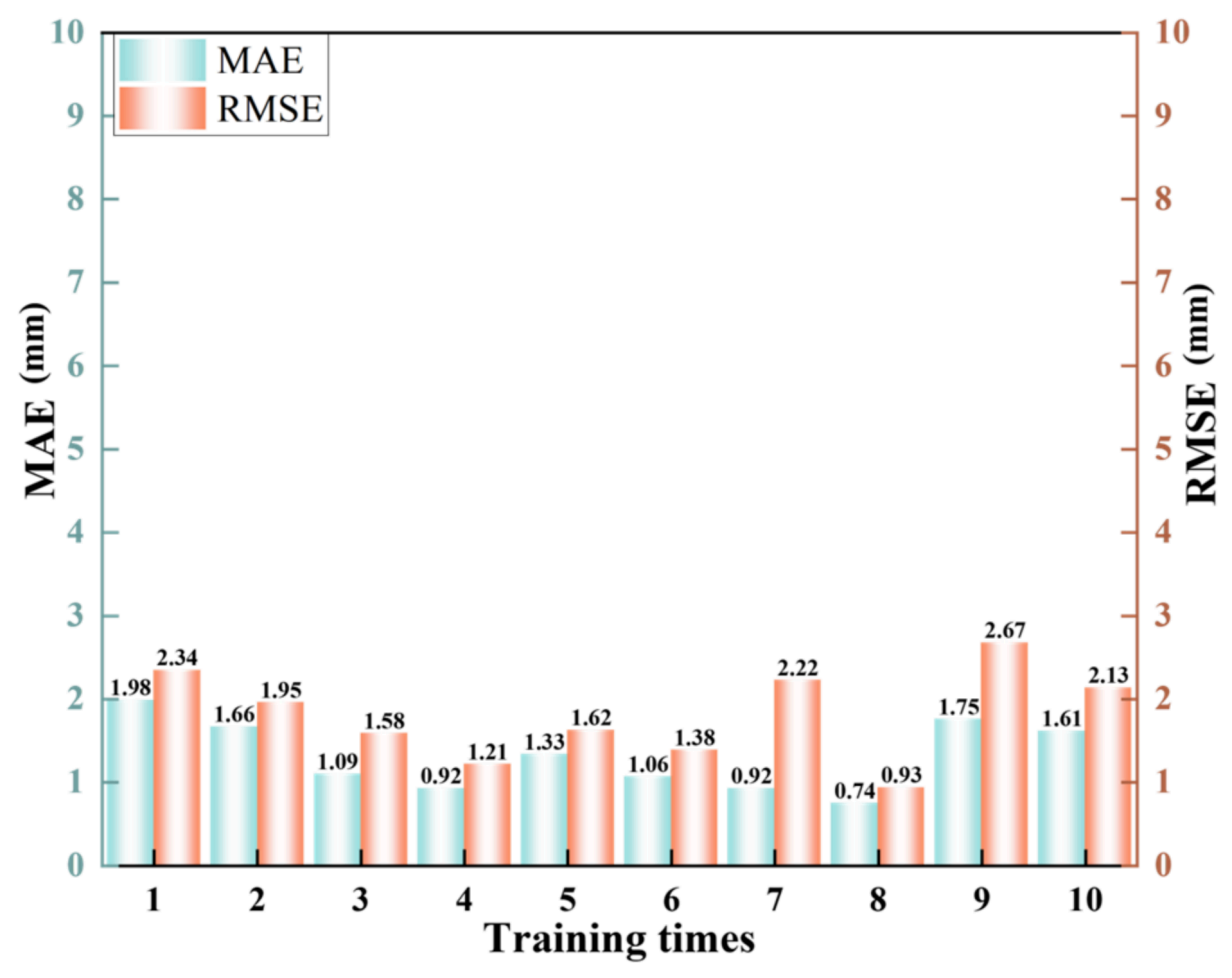
Damage quantification index of the proposed method under transfer scenario 1.

In order to verify the stability of the model in the damage size quantization process and reduce the influence of random fluctuations in the single test results, the damage quantization model was trained ten times independently. The damage quantization results on the test data set obtained under transfer scenario 1 are shown in Figure 9, where the horizontal axis represents the *i*-th training instance of the model and *i* is an integer ranging from 1 to 10, and the vertical axis indicates the values of the two quantification metrics at the *i*-th training iteration.

The experimental results depicted in Figure 9 reveal the performance of the proposed model after ten independent training sessions under the cross-area transfer setting of scenario 1. Specifically, the MAE exhibited minor fluctuations, with a maximum value of 1.98 mm and a minimum value of 0.74 mm, both of which remained below 2 mm. This result indicates that despite the presence of error fluctuations, the model maintains an acceptable level of precision in damage quantification. Similarly, the results of RMSE showed a comparable trend, with a maximum value of 2.67 mm and a minimum value of 1.21 mm, with neither exceeding the error threshold of 3 mm. These results further confirm the robustness of the model in terms of damage quantification accuracy, especially when dealing with the cross-area damage diagnosis tasks of CFRP plates under small-sample conditions.

**Figure 10:**
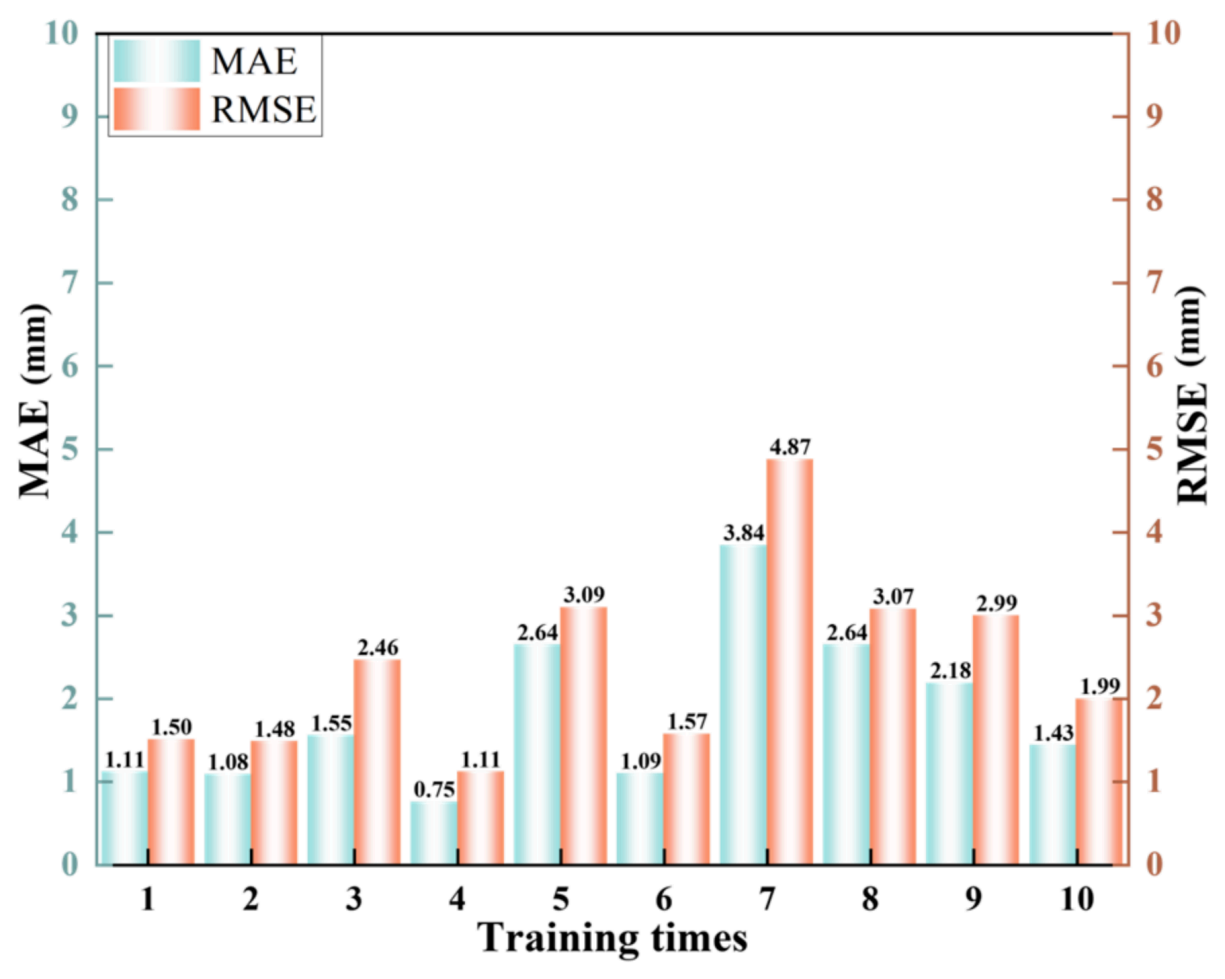
Damage quantification index of the proposed method under transfer scenario 2.

**Figure 11:**
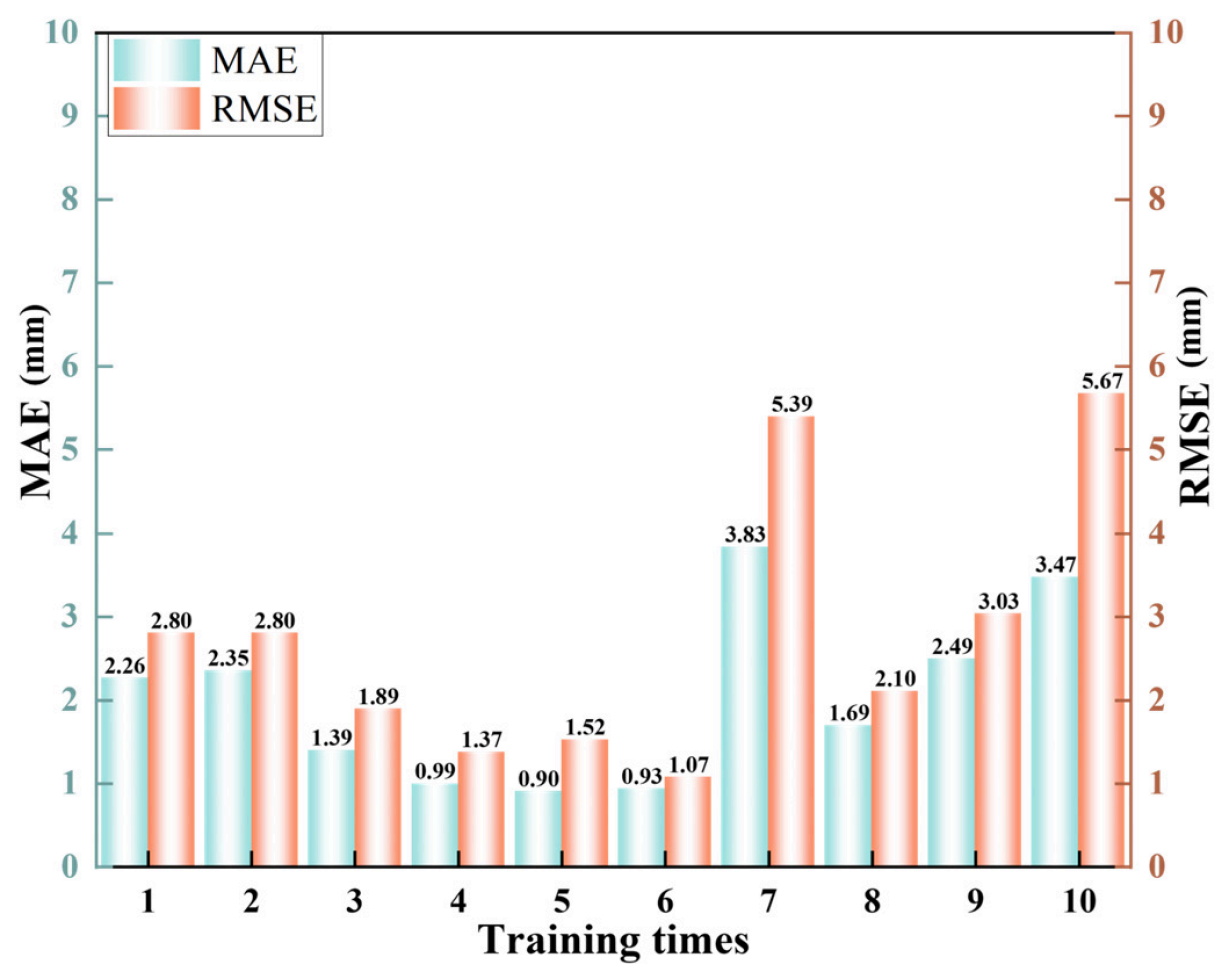
Damage quantification index of the proposed method under transfer scenario 3.

Furthermore, Figure 10 and Figure 11 respectively illustrate the results of cross-area damage quantification under transfer scenario 2 and transfer scenario 3. By comparing the damage quantification errors across these three transfer scenarios, it can be observed that areas closest to the source domain exhibit smaller damage errors. This phenomenon can be attributed to the fact that the target area close to the source area has higher similarity in the damage signal so the damage quantization error is smaller, which is logically reasonable. In summary, whether it is cross-area damage quantification under transfer scenario 1 or under transfer scenarios 2 and 3, the method proposed in this paper can accurately predict the size of the damage, which proves the robustness of the model.

### 4.2. Comparison with Other Machine Learning Methods

**Figure 12:**
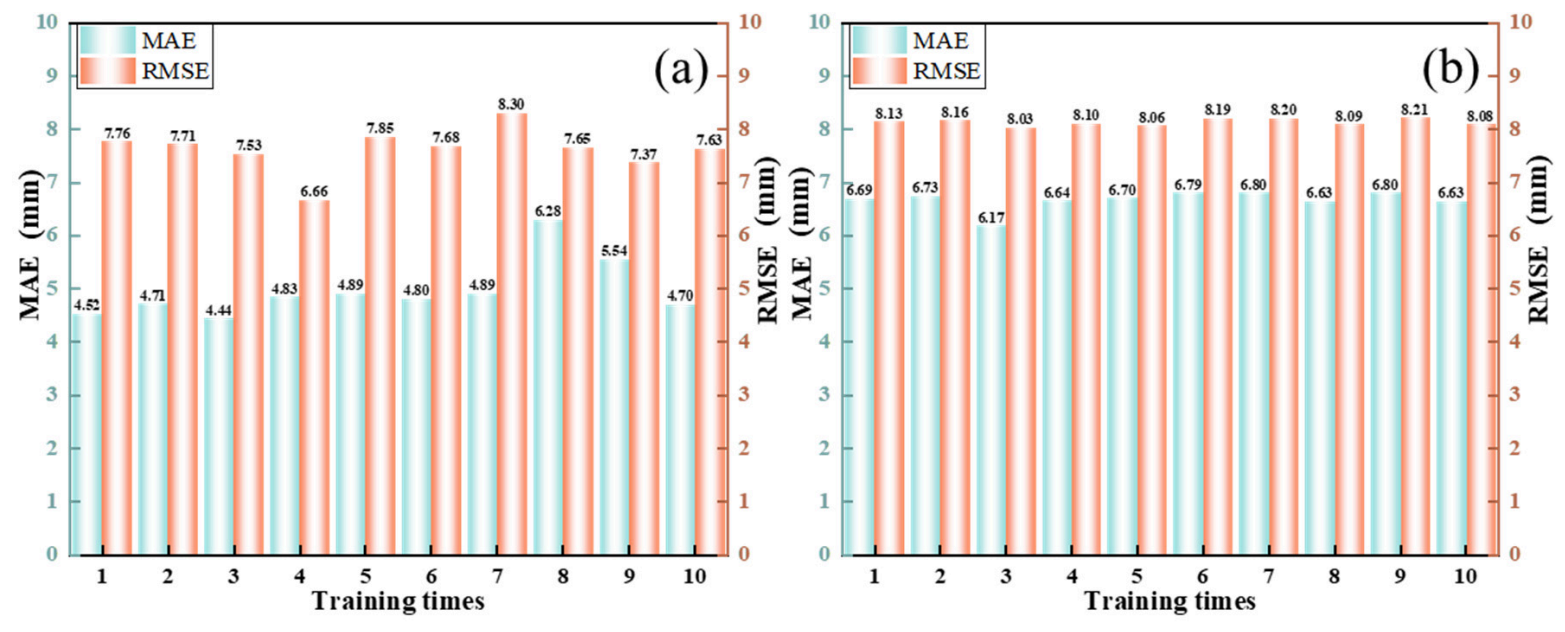
Damage quantification metrics of the comparison methods in scenario 1: (**a**) CNN damage quantification metrics; (**b**) Transformer damage quantification metrics.

**Figure 13:**
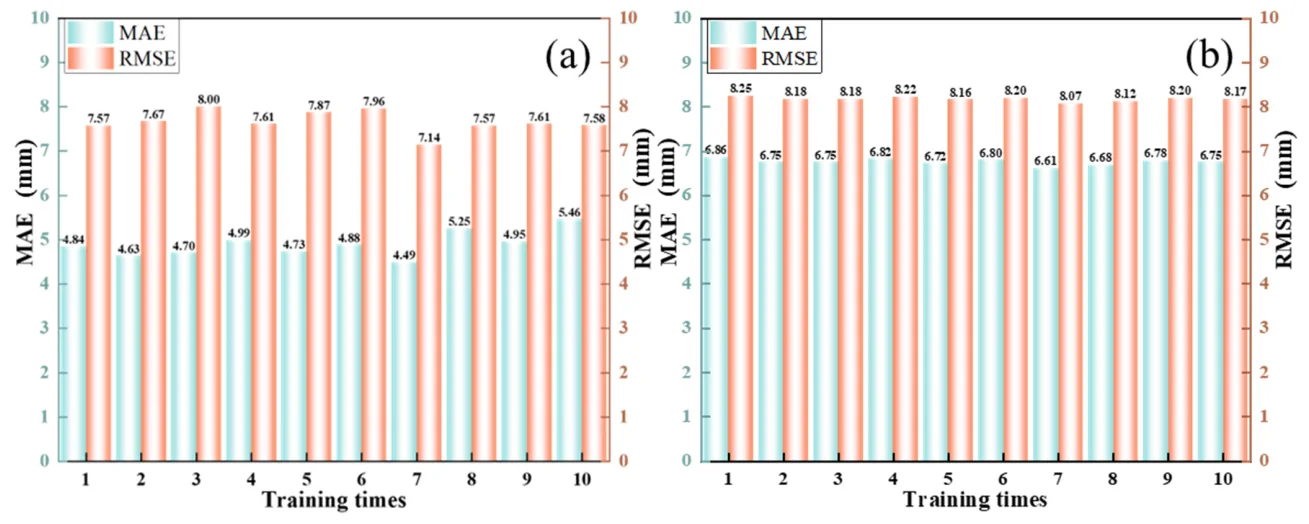
Damage quantification metrics of the comparison methods in scenario 2: (**a**) CNN damage quantification metrics; (**b**) Transformer damage quantification metrics.

**Figure 14:**
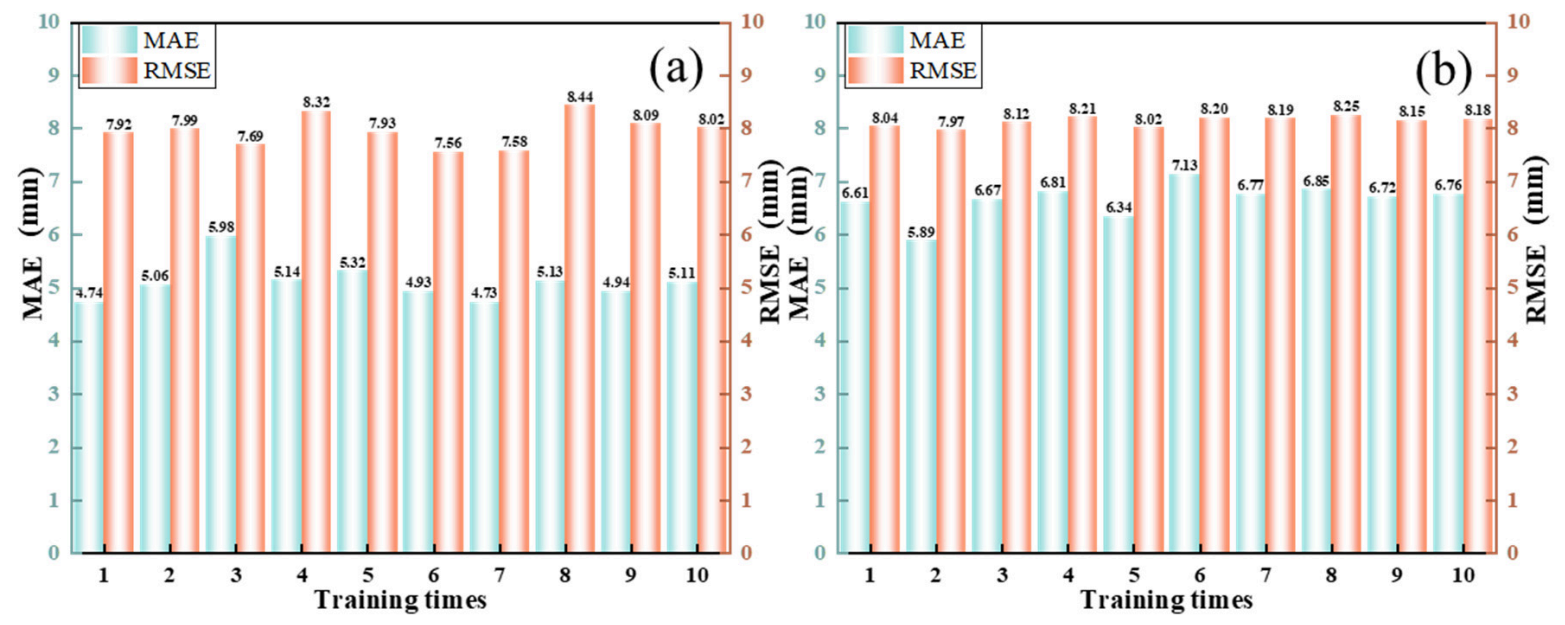
Damage quantification metrics of the comparison methods in scenario 3: (**a**) CNN damage quantification metrics; (**b**) Transformer damage quantification metrics.

To validate the superiority of the cross-area transfer method proposed in this study for damage quantification, we conducted comparative experiments with widely used damage quantification methods. Specifically, this study selected the classic deep learning method known as the Convolutional Neural Network (CNN) [47] and a deep learning architecture based on a self-attention mechanism called the Transformer model [48] as the control group. It should be noted that the proposed method incorporates a feature-based transfer learning mechanism with explicit adaptation to the target domain, which represents one of the key innovations of this work. In contrast, the CNN and Transformer models were implemented following their original formulations without integrating transfer learning principles, reflecting their standard application scenarios in damage quantification tasks. This comparison is intentionally designed to evaluate the practical advantage of the proposed cross-area transfer framework over conventional deep learning approaches under cross-domain conditions, where distribution discrepancies between source and target structural areas are present. The performance of each model was comprehensively assessed through three designed experiments, with MAE and RMSE serving as key metrics for evaluating model performance, and the values of these metrics were recorded in detail. The results of the comparative experiments are presented in Figure 12, Figure 13 and Figure 14, respectively.

The results demonstrate that the cross-area transfer model proposed in this paper shows excellent damage quantification ability. By comparing the results in Figure 9, Figure 10, Figure 11, Figure 12, Figure 13 and Figure 14, it can be observed that in three cross-area transfer scenarios, the performance of the CNN [35] and Transformer [36] models in damage quantification is inferior to the method proposed in this paper. The reason is that the two comparative methods failed to effectively extract features that are sensitive to damage propagation and insensitive to location, which are essential for transferability. The method presented in this study significantly improves the accuracy of damage quantification by extracting transferable features, thereby demonstrating superior performance in cross-area transfer scenarios.

To comprehensively assess the global performance of the damage quantification methods, this paper averaged the error quantification metrics over 10 trainings. The average error quantification results of the three methods across three different damage quantification transfer scenarios are summarized in Table 2. By analyzing the data in Table 2, it is evident that in three transfer scenarios, the method proposed in this paper exhibits lower average MAE values compared to the other two methods. This indicates that its predicted results are closer to the actual damage sizes overall, achieving more accurate predictions. Additionally, the lower average RMSE values of the MSACADQ-TL method further demonstrate that there are fewer extreme errors in predicting damage sizes, leading to more stable predictions. Therefore, in comparison to the CNN model and Transformer method, the MSACADQ-TL method shows greater robustness and generalization capability in the average damage quantification error assessment across the three transfer scenarios.

## 5. Conclusions

In this paper, an advanced transfer learning damage quantization model, the MSACADQ-TL method, is introduced for CFRP structures, especially in the small-sample data environment. The model relies on the interaction between the damage signal and the damage size of the Lamb wave to develop an innovative multi-scale damage feature extraction framework. The advantage of this framework is that it can integrate the multi-dimensional damage features of frequency domain and time domain and extract the transferable damage features through the feature mapping technology. In addition, by combining the artificial feature extraction method with deep neural networks, the model refines features that are sensitive to damage, thereby enhancing the ability of the model to express the damage features. The model is not only capable of effectively learning damage features and accurately quantifying damage sizes with a small amount of labeled data but also achieves cross-area damage quantification without the need for new damage area samples to participate in model training. It is especially crucial for damage quantification tasks in large-sized CFRP structures or complex environments, providing a more intelligent and efficient solution for CFRP structural health monitoring.

## Figures and Tables

**Table 1:** Three cross-area damage quantification scenarios.

Cross-Area Transfer Scenario	Source Domain Area	Target Domain Area
Transfer scenario 1	Locations 1 and 2	Locations 3 and 4
Transfer scenario 2	Locations 1 and 2	Locations 5 and 6
Transfer scenario 3	Locations 1 and 2	Locations 7 and 8

**Table 2:** The mean error quantification metric.

Method	Transfer Scenario 1		Transfer Scenario 2		Transfer Scenario 3	
	M-MAE (mm)	M-RMSE (mm)	M-MAE (mm)	M-RMSE (mm)	M-MAE (mm)	M-RMSE (mm)
CNN	4.96	7.61	4.89	7.66	5.11	7.96
Transformer	6.66	8.12	6.75	8.17	6.65	8.13
OUR	1.30	1.80	1.83	2.41	2.03	2.76

## Data Availability

The raw data supporting the conclusions of this article will be made available by the authors on request.
